# Does the Effort Meet the Challenge in Promoting Low-Carbon City?—A Perspective of Global Practice

**DOI:** 10.3390/ijerph15071334

**Published:** 2018-06-25

**Authors:** Yingli Lou, Liyin Shen, Zhenhua Huang, Ya Wu, Heng Li, Guijun Li

**Affiliations:** 1 School of Construction Management and Real Estate, International Research Center for Sustainable Built Environment, Chongqing University, Chongqing 400045, China; 20160302057t@cqu.edu.cn (Y.L.); 20150302017t@cqu.edu.cn (Z.H.); mswuya@126.com (Y.W.); 2 Department of Building and Real Estate, Hong Kong Polytechnic University, Hong Kong 999077, China; bshengli@polyu.edu.hk; 3 Center of International Cooperation Research, Central University of Finance and Economics, Beijing 100081, China; ligj@cufe.edu.cn

**Keywords:** low-carbon city, carbon emissions, emission reduction policy, global perspective, climate change, sustainability

## Abstract

Global warming caused by carbon emissions has been recognized as a challenge to human sustainable development, and low-carbon city development is widely considered as an effective strategy to address this challenge. Numerous emission reduction measures have been implemented, and considerable efforts have been devoted in promoting low-carbon city. This paper examines whether sufficient efforts have been paid to these typical emission sectors, including Building, Industry, Energy Transformation, and Transportation by referring to the shared responsibility of each sector. The shared responsibility of individual emission sector is calculated by applying energy consumption data in 2014 World Energy Balance. The efforts contributed in emission reduction by each sector are examined by analyzing the low-carbon city work plans of 24 representative sample cities, which are selected globally. The research results demonstrate that sufficient emission reduction efforts have been paid in the Building sector and Transportation sector. But the Industry sector and Energy Transformation sector are less-attended in addressing emission reduction. The reason for the sufficient efforts paid in the Building sector and Transportation sector is considered as that the efforts for emission reduction in these two sectors can bring more co-benefits. However, emission reduction in Industrial sector is generally considered to have the effects of holding back economic growth, and the emission reduction in the sector of Energy Transformation will need enormous investment for advanced technologies. Policy for emission reduction in the Industry sector and Energy Transformation sector is indispensable to promote low-carbon city. This study appeals that (1) low-carbon city can be effectively implemented only if carbon reduction policy is adopted to all industrial activities; (2) multiple channels of financial resources should be established to support cities to mitigate carbon emissions in Industry sector; (3) cooperation on the development of clean energy technology between cities should be promoted; and (4) efforts should be paid to reduce carbon emission from using traditional energy transformation equipment by improving their efficiency.

## 1. Introduction

Sustainable development is confronted with a great challenge from global warming. It has been widely reported that global warming has become increasingly severe particularly in recent years [1]. The consequence of global warming has caused multiple repercussions on the environment, natural resource, and human well-being [2,3,4,5,6,7]. For example, one of the most serious consequences of global warming is sea level rise [8], which causes the submergence of coastal land. Thus, many countries and cities are in the danger of disappearing. It was reported that Kiribati has already purchased land to move its entire population [9]. Another serious consequence of global warming is the warming ocean [10], which increases the frequency and intensity of storms and other weather events. It was reported that over 600,000 people died and 4.1 billion people wounded in weather-related events over the last two decades, inducing economic costs in excess of $1.9 trillion [11]. Therefore, tackling global warming is one of the most important and urgent issues for human sustainable development.

Scientists have almost unanimously concluded that the only way of effectively forestalling global warming in the long run is to reduce the emissions of greenhouse gases, chief among which is carbon dioxide [12]. Emissions of carbon dioxide are largely from the burning of fossil fuels in cities [13], which is ubiquitous in all sectors including the industry, transportation, and domestic sectors of economies. With this recognition, international conferences on carbon emission reduction have been organized continuously since 1979, when the first global climate change conference took place in Geneva. Various guidelines for mitigating carbon emissions have been promulgated by international agencies, such as World Bank (WB), World Resources Institute (WRI), C40 Cities Climate Leadership Group (C40), United Nations Human Settlement Programme (UN-Habitat), and United Nations Environment Programme (UNEP). Researchers have contributed considerable efforts in finding out solutions for emission reduction. In practice, an increasing number of cities at global level have been formulating policies to reduce the carbon emissions. According to the report by C40 [14], 91 major cities have joined C40 cities, which contribute to 25% of global GDP. Many cities have defined low-carbon city mission in their cities’ development blueprints, for example, 1050 cities in the United States, 40 cities in India, 100 cities in China, and 83 cities in Japan, as reported by Gomi, Shimada, & Matsuoka [15]. These cities have started to implement various low-carbon programs in the sectors of building, industry, and others.

However, it appears unclear whether we have done what we should do in promoting low-carbon city and whether sufficient efforts are given to these sectors which release more emissions in practice. It is generally considered that the major emission sectors in a city have more potential to reduce carbon emissions [16,17], and they should give more efforts to reduce emissions [18]. Without sound examining whether efforts are properly and sufficiently given, cities will not only be unable to achieve the low-carbon city goal, but also waste resources invested [19]. It is therefore essential to find out whether the efforts are sufficient in each emission sector to tackle carbon emissions. This understanding will enable city governments to formulate effective measures to reduce carbon emissions in these major emission sectors which not give sufficient efforts.

The existing research works in the context of low-carbon city practice can be classified as empirical studies of individual cities, case-based studies of various cities, and evaluation on low-carbon city performance. In referring to the city of Bangkok as an empirical case study, Phdungsilp [20] analyzed 16 proposed carbon reduction policies, and the results demonstrate that the most significant carbon reduction policies are in the transport sector. Lo [21] found the reasons for poor performance in carbon reduction in referring to the empirical study of Changchun, including poorly designed evaluation system, loosely defined reduction targets, and the lack of reliable statistics on energy consumption. Liu and Qin [22] decomposed low-carbon city policies into three elements: goal, contents, and instruments through archival analysis on official documents and field interviews across 10 Chinese cities. Based on the carbon emission status of 30 Chinese cities, Lynn et al. [23] proposed a low-carbon indicator system for China. Zhou et al. [24] evaluated 36 global cities on the performance of carbon reduction by using the DPSIR (Driving forces-Pressures-State-Impacts-Responses) causal-effect framework. Wu et al. [25] evaluated 284 Chinese cities on the performance of carbon emissions and classified these cities into four types, including low-carbon city, relatively low-carbon city, relatively high-carbon city, and high-carbon city.

There are two limitations noted in the existing studies. First, little research is conducted from a global perspective. In fact, the emission problem threatens all human beings, and mitigating emissions requires global efforts. It is therefore essential to examine low-carbon city practice from a global perspective [24]. Second, there is little research on whether the major emission sectors are effectively addressed with sufficient efforts. In other words, it is unknown whether the major emission sectors have been put on sufficient emission reduction measures. Only these major emission sectors from all cities participate in the mission of low-carbon city practice and contribute sufficient efforts, the emission reduction can be achieved globally. Therefore, the aim of this study is to identify those less-attended emission sectors where more efforts should be paid.

The remainder of this paper is organized as follows: Section 2 introduces the methods used in this study. Section 3 classifies carbon emission sectors. Section 4 calculates shared responsibility between individual emission sectors. Section 5 investigates the efforts contributed in emission reduction between emission sectors. Discussion and policy implication of the research findings are provided in Section 6, followed by the conclusion section.

## 2. Research Framework and Methods

In order to achieve the aim of this study, the following research works are planned:

(1) Carbon emission sectors will be classified as a basis to examine the practice of low-carbon city;
(2) The shared responsibility of individual emission sector is calculated to determine the level of efforts each sector should contribute;
(3) The contributed efforts by individual emission sector is analyzed to figure out whether the efforts are sufficient.

**Figure 1:**
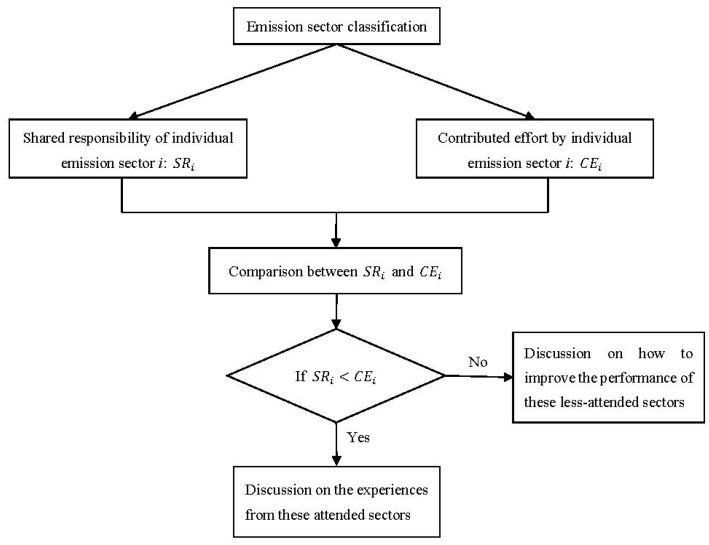
Research framework.

The procedures of these research works can be presented graphically, as shown in Figure 1.

Carbon emission sectors will be classified through conducting comprehensive literature review. As this research aims to examine the implementation practice of low-carbon cities from a global perspective, it is important that the classification of carbon emission sectors is adaptable globally. Therefore, literature and global guidance for carbon emission reduction will be referred for the classification of these sectors.

Secondly, the shared responsibility of emission sector *i* (*SR_i_*) is calculated according to formula (1).

(1) $$\;SR_{i}=\frac{C_{i}}{\sum_{i=1}^{m}C_{i}}\;i=1,\;2,\;3,\;\ldots \;m$$

It assumed that there are *m* emission sectors. *C_i_* is the carbon emissions released by sector *i*, which is calculated based on the method provided by Intergovernmental Panel on Climate Change (IPCC). This method is widely used by researchers to calculate carbon emissions from the perspective of energy consumption [26,27,28,29]. The method is expressed in the following formula:

(2) $$\;C_{i}=\frac{44}{12}\overset{k}{\underset{j=1}{\sum }}K_{j}\times E_{ij}\;i=1,\;2,\;3,\;\ldots \;m\;$$

**Figure 2:**
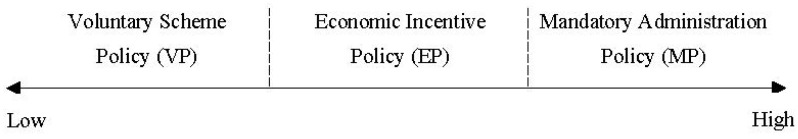
The degree of policy enforcement by government.

In applying this method, it is assumed that there are *k* types of energy. For a specific emission sector *i*, the consumption on energy *j* is *E_ij_*, which needs to be converted to standard coal equivalent in applying model (2). The expression $\frac{44}{12}$ in the formula (2) represents the molar ratio of carbon dioxide to carbon atom. *K_j_* is the carbon emission factor of energy *j*. In the third part of this research, efforts contributed in emission reduction by individual sector *i* will be obtained through examining the emission reduction policies adopted within the sector. These policies can be identified from examining literatures and official documents. In this study, a group of sample cities are selected globally. It is considered that major policies will be included in these city plans for promoting low-carbon city. These plans include various emission reduction policies, which are specified in implementation measures and terms in various sectors, such as building and transportation. Based on the identification of the emission reduction policies designed for each emission sector, the efforts contributed by a concerned sector will be evaluated from three perspectives, namely, the number of policies designed for the sector, the enforcement degree of a specific policy, and the number of cities that adopt the policy. The contributed efforts by sector *i* (*CE_i_*) can therefore be evaluated through the following formula:

(3)  CEi=CEi'∑i=1mCEi' i=1, 2, 3… m

(4) $$\;CE_{i}^{'}=\overset{n}{\underset{j=1}{\sum }}D_{ij}\times N_{ij}\;i=1,\;2,\;3\;\ldots \;m$$

where *n* represents the total number of emission reduction policies introduced for emission sector *i*. *N_ij_* represents the number of cities adopting the policy *j* in their work plans for emission sector *i. D_ij_* represents the enforcement degree of the policy *j* in sector *i*. *D_ij_* can be classified into three levels according to the principle of policy instrument and the references provided by the World Bank (WB) and the Organization for Economic Co-operation and Development (OECD) [30,31], as shown in Figure 2.

In the enforcement framework, Mandatory Administration Policy (MP) is taken by the government departments, which leads to carbon emission reductions directly. Economic Incentive Policy (IP) is issued by government departments, which stimulates organizations and public to reduce carbon emissions by receiving economic compensation or penalty. Voluntary Scheme Policy (VP) is proposed by government departments as reference guidelines to promote carbon emission reduction in a society. In using this framework, the value of *D_ij_* is designed by considering that a higher enforcement degree will request for more efforts, and vice versa. According to this analogy, *D_ij_* is given with a value of 3 if a concerned policy is Mandatory Administration Policy, a value of 2 if the policy is Economic Incentive Policy, and a value of 1 if the policy is Voluntary Scheme Policy. 

For examining whether the contributed efforts are sufficient in each emission sector to promote low-carbon city, the comparison will be conducted between the shared responsibility of individual emission sector *i*, *SR_i_*, in formula (1) and the contributed efforts by the sector, *CE_i_*, in formula (3). 

## 3. Carbon Emission Sectors

The existing research works have presented different types of classifications of carbon emission sectors. In analyzing the distribution of emission reduction potentials, Akimoto et al. [32] identified six carbon emission sectors, including power, industries, transportation, residential & commercial, agriculture, and waste. The study by Alcantara and Padilla [33] presents five major emission sectors, including building, domestic transport, chemical, food, and restaurants and hotels. Lynn et al. [23] proposed a classification of carbon emission sectors based on energy end-user, including industry, residential, commercial, transport, and electric power. Whilst the sources of carbon emissions are multiple, they can be classified into two categories: emissions from energy consumption and that from non-energy consumption activities. Energy consumption includes the consumption on coal, oil, natural gas, nuclear power etc. These consumptions will generate carbon emissions, called energy consumption emissions. On the other hand, there are non-energy consumption activities that can also generate emissions, such as chemical or physical transformation of material, disposal of waste, and the respiration of plants and animals. It is commonly appreciated that energy consumption is the dominant source of carbon emissions, whilst non-energy consumption emissions have limited reduction potential [34,35,36]. Therefore, the carbon emission sectors referred in this study are classified from the perspective of energy consumption.

**Figure 3:**
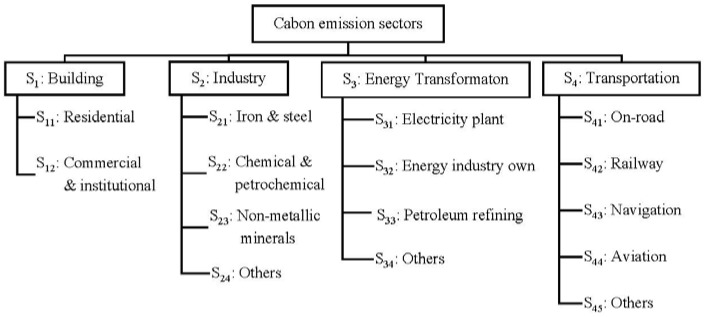
Classification framework of emission sectors.

Classification of emission sectors has been addressed in various indicator systems introduced for guiding the practice of low-carbon city by various international organizations. For example, the United Nations Human Settlements Program issued the Planning for Climate Change [37]. The International Energy Agency issued the World Energy Balance [38]. The World Resources Institute, C40 Cities, and the International Council for Local Environmental Initiatives jointly issued the Global Protocol for Community-Scale Greenhouse Gas Emission Inventories [39]. In referring to these international guidelines, four carbon emission sectors are classified, including Building, Industry, Energy Transformation, and Transportation, with each composing of a number of sub-sectors. Figure 3 presents a framework of emission sectors, which will be used as a basis for analysis in this study.

In Figure 3, the carbon emission in Building sector is generated by residential buildings, commercial and institutional buildings. Industry emission sector is specified by sub-sectors as listed in the International Standard Industrial Classification of All Economic Activities (ISIC) [40]. Energy Transformation comprises the conversion of primary forms of energy to secondary and further transformation (e.g., coking coal to coke, crude oil to oil products, and fuel oil to electricity). Transport sector refers to all types of transport activities (in mobile engines) across all economic sectors.

## 4. Shared Responsibility of Individual Emission Sectors

The shared responsibility of individual emission sector *i* will be calculated by applying the formulas (1) and (2). For conducting the calculation, the data of the consumption on various types of energy by all individual sectors listed in Figure 3 need to be collected, which can be calculated according to the 2014 World Energy Balance (WEB) report. In WEB, there are 37 sectors related to this research, and their energy consumption data have also been provided, as shown in the Appendix Table A1. These 37 sectors are aggregated into 15 sectors as shown in Figure 3 according to the principle of industry combination [41,42]. The details of the aggregation are shown in Table 1.

By referring to the data in Table A1 and Table 1, the energy consumption data about all the aggregated sectors can be calculated, as shown in Table 2.

It is noted in Table 2 that there are negative values in the sectors S_31_, S_33_, and S_34_. This is because these sectors not only consume energy, including coal, crude oil, natural gas, and biofuels, but also generate energy, including oil products, heat, and electricity. The emissions from energy consumptions are offset by the energy generated.

The carbon emission factors for energy in Table 2 are quoted from the research by Wang and Ye [29]. However, the emission factors of oil products and biofuels are not offered in the reference [29] and will be calculated indirectly. In fact, emission factors of oil products and biofuels are given in the 2006 IPCC Guidelines for National Greenhouse Gas Inventories [43] but are measured in different calculation units. Therefore, a conversion coefficient will be used to convert the values of the emission factors given in [43] to the values in line with the reference [29]. For obtaining the conversion coefficient, a product (for example, crude oil) for which its emission factor is available both in [29] and [43] needs to be referred. In this case, the emission factor of crude oil in [29] is 0.5857 tC/tce, and that in [43] is 73.3 tC/TJ. It can be seen the calculation units in the two references are different. The conversion coefficient between the value offered in [29] and that in [43] can be calculated: 73.3/0.5857 = 125.1 (tce/TJ). In other words, the conversion coefficient for converting the values of the emission factors measured in IPCC [43] to the value in line with the reference [29] is 125.1 tce/TJ. With this conversion coefficient, the emission factors of oil products and biofuels can be obtained.

There are 13 major types of oil products and 10 types of biofuels [38], as presented in Table 2. The emission factors for these individual types of products are available in IPCC [43]. The emission factor of oil products can be measured by the average value of the 13 kinds of oil products, which is 71.0 tC/TJ. Similarly, the emission factor of biofuels is measured by the average value of 10 biofuels [38], which is 80.4 tC/TJ. These two values now can be converted to the values measured in the way adopted in the reference [29], with the results of 71.0/125.1 = 0.5675 tC/tce and 80.4/125.1= 0.6427 tC/tce, respectively. These two values, together with other values for all types of energy in [29] will be used for further analysis, as shown in Table 3. 

By applying the data in Table 2 and Table 3 to formula (2), carbon emissions released by various sectors can be obtained, as shown in Table 4, 

By applying the data in Table 4 to formula (1), the shared responsibility between four emission sectors can be obtained, as shown in Table 5.

**Figure 4:**
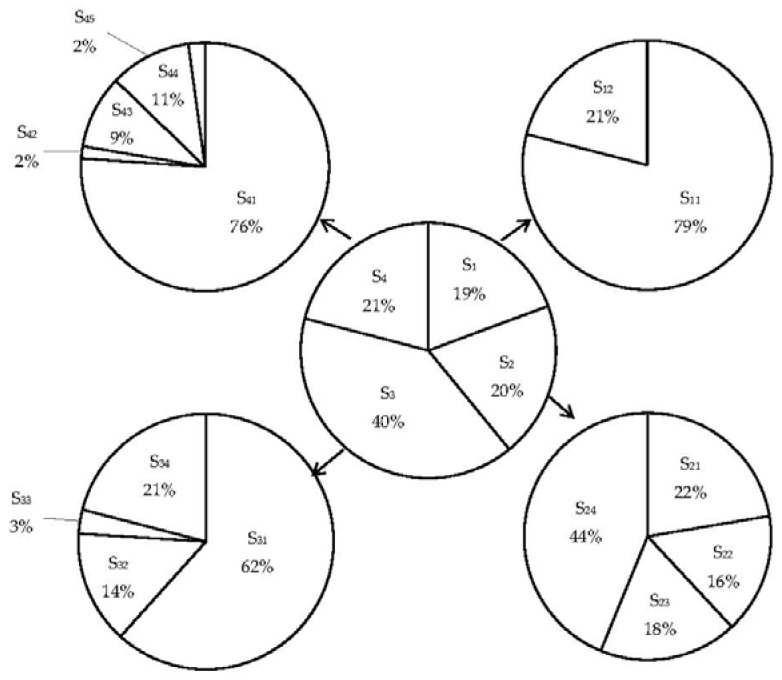
Shared responsibility between various emission sectors.

The shared responsibility between sub-sectors within each emission sector can also be calculated by using the same method defined in formula (1), and the results are shown in Table 6. The data in Table 5 and Table 6 can be further presented graphically, as shown in Figure 4.

As shown in Figure 4, the shared responsibilities in the sectors of Building (S_1_), Industry (S_2_), Energy Transformation (S_3_), and Transportation (S_4_) are distributed in 19%, 20%, 40%, and 21%, respectively. S_3_ accounts for the largest shared responsibility. This is because that the conversion of primary forms of energy to secondary or further energy forms consumes huge amount of energy and produces a vast amount of carbon emissions. Therefore, exploring appropriate carbon reduction policies in the sector S_3_ (Energy Transformation) is considered very important to achieve carbon abatement globally. In particular, the policy for carbon reduction in the process of producing electricity should be adopted, as it can be seen from Figure 4 that electricity plants (S_31_) is the major component of S_3_. 

Figure 4 shows that S_1_ (Building) is another major emission sector. Within this sector, the subsectors Residential (S_11_) and Commercial & institutional (S_12_) account for 79% and 21% respectively, suggesting that carbon emissions of residential building are much more than that of commercial & institutional building. Therefore, reducing energy consumption in residential building deserves more efforts. 

Figure 4 also demonstrates that S_2_ (Industry) is another significant emission sector, contributed by Iron and Steel (S_21_), Chemical and Petrochemical (S_22_), Non-Metallic Minerals (S_23_), and Others (S_24_), as shown in Figure 4. It can be seen that all industrial sectors have a share to the emission generation. Therefore, policies for reducing emissions in conducting all industrial activities should be explored.

In referring to the emission sector S_4_ (Transportation), the subcomponent of on-road (S_41_) is the biggest emitter. On-road transportation generally includes cars, taxis, electric bicycles and buses, and measures for reducing emissions generated from on-road transportation should be taken.

## 5. Contributed Effort by Individual Emission Sectors 

The efforts contributed in emission reduction by the four classified emission sectors (S_1_, S_2_, S_3_, S_4_) will be analyzed in this section. The data used for the analysis are retrieved from examining sample cities’ low-carbon work plans.

### 5.1. Selection of Sample Cities

The sample cities are selected from these main carbon emission countries and regions, including China, the United States, the European Union, India, the Russian Federation, and Japan. It was reported that the carbon emissions from these countries and regions was more than 65% of the whole world carbon emission since 2004 [44], with the data in Table 7. Therefore, it is considered that carbon emission reduction in these countries will make significant contribution to the total emission reduction globally.

As there are many cities engaging low-carbon practice in these referred countries, the selection of sample cities is based on two criteria: (1) those on the C40 list where all members have been actively practicing low-carbon city development and (2) those cities where relevant data are publically available. As a result, 24 cities are selected, as shown in Table 8. It is realized that the time frames of the work plans for different cities are different, some even like historical data. But, cities promulgate their work plans in different years and update the work plan in different intervals. These data in Table 8 are the most updated work plans obtainable in this study.

Generally, all the work plans from different nations have covered all four emission sectors, namely, Building, Industry, Energy Transformation, and Transportation. However, different nations have different issues to focus. For example, cities in developing countries put more weights on Industrial sectors, whereas the cities in developed countries give more concern to Building and Transportation.

### 5.2. Contributed Efforts

According to the research method described in the Section 2, contributed efforts between various emission sectors will be analyzed by examining to what extent the emission reduction policies introduced are actually adopted in cities’ work plans. The typical emission reduction policies are defined in various guidelines issued by international organization and researchers. These guidelines are listed in Table 9.

A cluster of carbon reduction policies against four categories of emission sectors are retrieved from the guidelines specified in Table 9. The classification for a specific emission reduction policy between Mandatory Administration Policy (MP), Economic Incentive Policy (EP), or Voluntary Scheme Policy (VP) is drawn according to the method defined in Figure 2. For example, the policy of energy efficiency performance standards in new building for Building sector is a mandatory policy MP, because efficiency performance standards must be promulgated and executed by relevant government departments. As a result, three types of carbon reduction policies (MP, EP, VP) against four categories of emission sectors are retrieved, summarized in Table 10.

The number of cities that adopt the specific policies is counted by examining these sample cities’ work plans listed in Table 8. First, this counting process is conducted by individual research team members. In the case where the expression of a specific policy is not consistent between the policy specification by cities and that specified in Table 10, group discussion is organized to reach consensus. The results of the calculation are shown in Table 11, indicating the number of cities in applying specific emission reduction policies in their city work plan.

**Figure 5:**
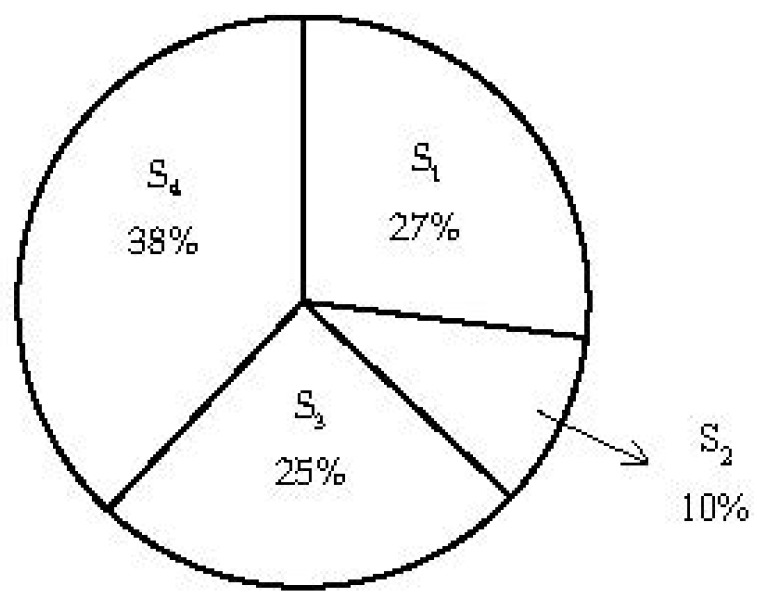
Distribution of contributed efforts in various emission sectors.

The contributed efforts in various sectors can be evaluated by applying the data in Table 11 to formulas (3) and (4), and the results are shown in Table 12 and Figure 5.

**Figure 6:**
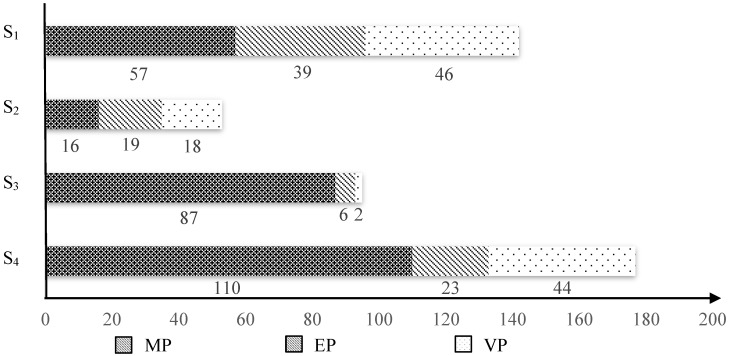
The number of the three kinds of carbon reduction policies adopted in four emission sectors.

Furthermore, according to the information in Table 11, the extent of applications of the three kinds of carbon reduction policies by four emission sectors can be obtained, as shown in Table 13 and Figure 6. 

It can be observed from Figure 5 that the contributed efforts by sector S_4_ (Transportation) accounts for much more than that for other sectors. In other words, the policies adopted for addressing emission reduction in Transportation sector account for large proportion. It can be seen from Table 13 and Figure 6 that the total number of policies adopted for reducing emission in S_4_ is 177. The reasons for this are not only because of the large number of policies available for this sector, but also because of the enforcement of policy application. Those popular enforced policies among sample cities are S_4_-MP_1_ (Transit-oriented transportation planning), S_4_-MP_10_ (Standards of vehicle fuel using efficiency), and S_4_-MP_13_ (Improving walk and bicycle path environment), as shown in Table 11.

Figure 5 also demonstrates that the contributed efforts by S_1_ (Building) and S_3_ (Energy Transformation) accounts for significant proportions, which are 27%, 25% respectively. This indicates that a reasonable number of policies for these two sectors have been adopted. Although the total number of policies adopted for reducing emission in S_3_ is smaller than that in S_1_, there are more Mandatory Administration Policies (MPs) adopted in S_3_, as shown in Figure 6. Therefore, the efforts contributed in S_1_ and S_3_ are both relatively good.

It is interesting to note that the efforts contributed by sector S_2_ (Industry) accounts for a small proportion. In referring to Table 10, there are a number of emission reduction policies available for S_2_, but the number of cities that adopt these policies is small.

## 6. Discussion and Policy Implications 

**Figure 7:**
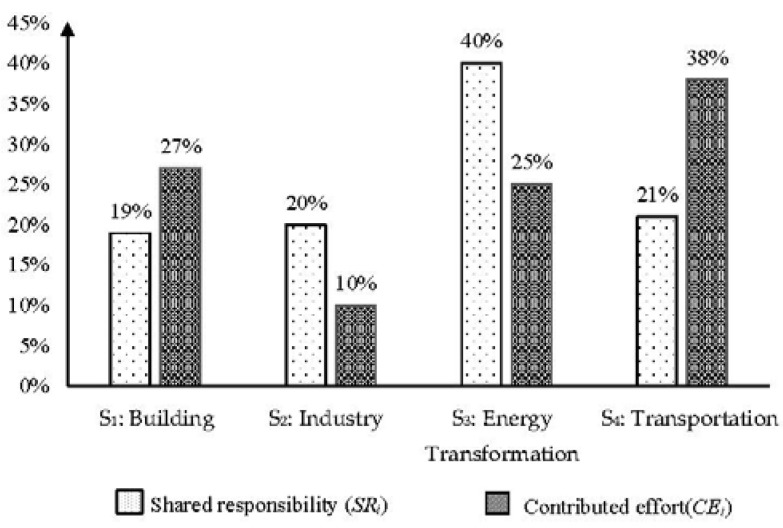
Gaps between shared responsibility and contributed effort.

Comparative discussions will be conducted between the shared responsibilities and contributed efforts in referring to individual emission sectors. The purpose of the comparison is to demonstrate whether the efforts contributed by each emission sector are sufficient in promoting low-carbon city. By using the data in Table 5 and Table 12, the gaps between the shared responsibility (*SR_i_*) and contributed efforts (*CE_i_*) across four emission sectors can be obtained, as shown in Figure 7.

**Figure 8:**
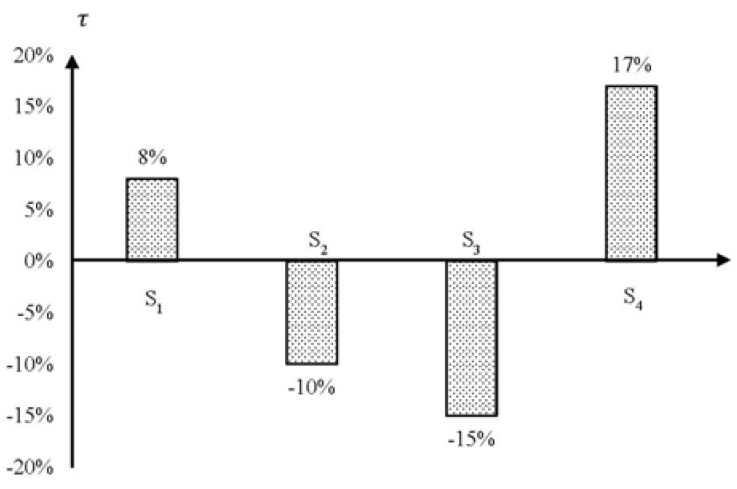
Level of efforts sufficiency.

Figure 7 tells that the efforts contributed in sector S_1_ and S_4_ are more than their responsibilities, whist the efforts by S_2_ and S_3_ are less than their corresponding responsibilities. Based on the information in Figure 7, the level of efforts sufficiency, denoted as τ, between four emission sectors can be found, as shown in Figure 8.

It can be observed from Figure 8 that S_1_ (Building) and S_4_ (Transportation) are well attended sectors in terms of the level of efforts sufficiency. However, S_2_ (Industry) and S_3_ (Energy Transformation) are less-attended.

### 6.1. Attended Emission Sectors

Figure 8 demonstrates that S_4_ (Transportation) is a significantly attended sector with a high positive value of τ. The reason why emission reduction in Transportation sector is favored is that the improvement of emission reduction in this sector can produce more co-benefits in achieving both climatic and other environmental goals simultaneously [76,77]. Furthermore, development of low-carbon transport system, for example walking track and biking, will improve walkability and mobility throughout the community. In this way, people can save time from congested roadways, and accident-related injuries can be reduced as well [78,79]. Therefore, emission reduction in transport system has been given with priority by governments through adopting more effective policies.

Furthermore, it appears that the effectiveness of emission reduction policies in S_4_ can be observed in short time. For example, “Transit-oriented transportation planning (S_4_-MP_1_),” “Standards of vehicle fuel using efficiency (S_4_-MP_10_),” “Improving walk and bicycle path environment (S_4_-MP_13_),” and “Parking fees (S_4_-EP_2_)” are all considered effective in many sample cities. Some cities have contributed great efforts in addressing emission reduction in transport system. For example, New York introduced 11 types of emission reduction measures in Transport sector for the aim of reducing 363.3 million metric tons carbon emissions from 2011 to 2030 [50]. Amsterdam has been promoting renewable-energy vehicles with the aim of powering 60 to 90% vehicles with green electricity generated by windmills, solar panels, and biomass power stations [61].

According to Figure 8, S_1_ (Building) is another attended sector in terms of emission reduction. It is widely appreciated that improving energy efficiency in Building sector can achieve a diverse set of community co-benefits, including reduction of pollutant emissions, increase of home value, and better security in energy appliances [52]. Typical carbon reduction policies aimed at energy saving in Building sector include S_1_-EP_4_ (Subsidies and tax credits for weatherization), S_1_-EP_5_ (Subsidies for purchasing energy-efficient equipment), and S_1_-VP_6_ (Encourage solar installation). Application of these energy saving policies can lead to the reduction of energy consumption and cost saving, thus the application can be supported and participated by citizens.

The significance of co-benefits from emission reduction policies has also been appreciated in previous studies. For example, Kousky and Schneider [76] pointed out that implementation of emission reduction policies is not driven primarily by public pressure, nor wholly for climate protection, but instead, by perceived co-benefits and cost savings.

### 6.2. Less-Attended Emission Sectors

Figure 8 demonstrates that sectors S_2_ (Industry) and S_3_ (Energy Transformation) are less-attended with negative values of τ. There are various reasons why the efforts for emission reduction in Industry sector is not sufficiently given in comparing to the shared responsibility by the sector. Industry sector involves a complex chain of activities, such as iron and steel, mining and quarrying, food and tobacco, and textile and leather. As different industrial activities have different production processes, it is more difficult to introduce mandatory reduction targets across all industrial activities. Usually, governments tend to focus on high-emission industries. For example, Beijing eliminated more than 3,000 high-emission industrial companies during 2010 to 2015 [45] with the aim of improving air quality. However, it is far from sufficient by only addressing emission produce among the high emission industrial activities. The efforts need to be contributed to all types of industry activities. For example, policies such as carbon tax relief (S_2_-EP_1_) and carbon trading (S_2_-EP_3_) can be introduced to all types of industrial activities to encourage emission reduction.

On the other hand, as the development of industry is one of the driving forces for economy growth particularly to developing countries, emission reduction in industrial production process usually is not positioned as priority. Other research works have also appreciated that countries in general focus more on the domestic interests of economic development instead of the global issue of carbon emission reduction [80]. There are a few cities which have contributed efforts in reducing emissions emitted from industrial activities, such as carbon cap-and-trade program in Tokyo [67], Shenzhen [48]. To encourage more cities to contribute efforts in industrial carbon reduction at global level, collaboration programs should be established. For example, the financial supports from developed countries and cities to those less developed countries for improving technologies in operating industrial activities.

Figure 8 also tells that sector S_3_ (Energy Transformation) is a significant less-attended emission sector. In fact, the contributed efforts by this sector is reasonably significant, as shown in Figure 7. However, the shared responsibility by this sector is much larger than that by the other sectors. The reason for the large shared responsibility by S_3_ is that emission generation from energy transformation is the major emission source. On the other hand, the room for contributing efforts in implementing policies to improve energy transformation in a specific city is limited, because the change of energy transformation mainly for electricity generation to clean-energy transformation will cost huge capital, which is not viable for many local governments. For example, the cost of wind power generation would be well over $1.3 billion to provide less than 1.5% of Hong Kong’s total electricity consumed [47], which cannot be afforded by many developing countries or cities. Therefore, cooperation is needed to develop clean energy between cities at global level.

It is good to note that many cities have been devoting efforts in developing clean energy instead of fossil fuels for electricity generation, such as solar energy and wind power. For example, among the 24 sample cities, 19 cities have launched solar power generation program, 16 cities have launched wind power generation program. Nevertheless, the efficiency improvement of traditional power plants is neglected to a large extent. For example, only four cities among the 24 sample cities have the program of improving power generation efficiency for reducing emissions. It is appreciated the improvement of Energy Transformation by replacing fossil fuels with clean energy requires more investment, and its effectiveness will be realized in a long time [81]. It is therefore considered that the practice of energy transformation dominated by fossil fuels will not be changed in a short time. However, the immediate emission reduction can be obtained through improving the efficiency of traditional power plants. Therefore, more efforts in applying policies to develop clean-energy power generation and improve efficiency of traditional power plants should be promoted collectively in order to achieve emission reduction in the sector of Energy Transformation.

## 7. Conclusions

The findings from this study show that, from a global perspective, the biggest carbon emitting sector is Energy Transformation, followed by Transport, Industry, and Building. The best effort contributor in addressing emission reduction is Transportation, followed by Building, Energy Transformation, and Industry. The sector of Building and Transport are well attended as the efforts contributed in these two sectors are more than their shared responsibilities. The emission sectors of Industry and Energy Transformation are less-attended as there are not sufficient efforts given in comparing to their shared responsibilities.

The findings provide important reference for governments to adopt effective reduction policies. The experience gained in the two good performers—namely, Building and Transport—can be promoted among cities or countries within global context. Less-attended sectors—namely, Energy Transformation and Industry—should be given more attention in order to achieve global carbon reduction. The lessons and difficulties encountered in the two poor performers should be surmounted in collaboration between cities.

The innovation and contribution of this study mainly lie in the following aspects. First, it provides a comprehensive understanding of global carbon emission composition, which is helpful to figure out which sectors should contribute more efforts in addressing emission reduction. Second, the holistic examination on low-carbon city policies provides governments with options on effective carbon reduction policies. Furthermore, the identification of less-attended emission sectors demonstrates the areas where should be contributed more efforts in order to achieve the mission of emission reduction. One typical limitation of this study is that the data obtained from IPCC, WEB, and the work plans of the sample cities in the study are not most updated. The further study is recommended when more updated data are available. Furthermore, investigating benchmarks for examining the performance of low-carbon city practice in referring to specific cities under different circumstances can be conducted in further research.

## Figures and Tables

**Table 1:** Aggregation of emission sectors.

Sector No.	Aggregated Sectors	Sectors in WEB
S_11_	Residential	E36
S_12_	Commercial & institutional	E37
S_21_	Iron & steel	E23
S_22_	Chemical & petrochemical	E24
S_23_	Non-metallic minerals	E26
S_24_	Others	E25, E27–E35
S_31_	Electricity plant	E3
S_32_	Energy industry own	E13
S_33_	Petroleum refining	E9, E10
S_34_	Others	E1, E2, E4–E8, E11, E12, E14
S_41_	On-road	E17
S_42_	Railways	E18
S_43_	Waterborne navigation	E20, E21
S_44_	Aviation	E15, E16
S_45_	Others	E19, E22

**Table 2:** Energy consumption in various sectors (*E_ij_*).

Sector No.	Coal	Crude Oil	Oil Products	Natural Gas	Biofuels	Heat	Electricity
S_11_	107.22	0.00	295.83	599.53	1,210.75	150.45	535.91
S_12_	49.96	0.00	122.15	259.61	34.99	50.36	437.94
S_21_	470.90	0.00	1.01	79.06	5.00	22.10	118.02
S_22_	142.00	0.09	78.57	172.95	2.33	71.77	117.34
S_23_	346.61	0.01	59.29	78.22	12.96	4.46	60.27
S_24_	266.93	9.62	272.08	310.53	256.18	77.42	548.70
S_31_	3018.60	58.03	288.42	1101.55	135.76	1.03	−2174.81
S_32_	145.37	16.31	293.28	416.71	19.91	49.73	203.14
S_33_	0.00	4090.03	−4016.98	0.00	0.00	0.00	0.00
S_34_	897.60	2024.75	−2005.13	596.91	217.25	−438.89	−11.19
S_41_	0.00	0.00	2663.84	54.43	104.46	0.00	0.31
S_42_	4.01	0.00	42.37	0.00	0.36	0.00	23.22
S_43_	0.00	0.00	354.29	0.16	0.73	0.00	0.00
S_44_	0.00	0.00	394.29	0.00	0.00	0.00	0.00
S_45_	0.07	0.00	11.49	84.39	0.01	0.00	3.60

Unit: million tonnes coal equivalent (The unit of electricity is 10^4^ million tonnes kWh); Data resource: International Energy Agency (IEA) [38].

**Table 3:** Carbon emission factors (*K_j_*).

Energy Type (*k*)	Coal	Crude Oil	Oil Products	Natural Gas	Biofuels	Heat	Electricity
Emission factor (tC/tce)	0.7559	0.5857	0.5675	0.4483	0.6427	0.67	0.272

The unit of carbon emission factor of electricity is tC/10^4^ kWh.

**Table 4:** Carbon emissions released by various sectors.

Emission Sector	S_1_: Building		S_2_: Industry				S_3_: Energy Transformation				S_4_: Transportation				
	S_11_	S_12_	S_21_	S_22_	S_23_	S_24_	S_31_	S_32_	S_33_	S_34_	S_41_	S_42_	S_43_	S_44_	S_45_
Carbon Emission (*C_i_*) (million tonnes)	5656	1462	1621	1140	1314	3178	9055	2105	425	3068	5879	123	739	820	166

**Table 5:** Shared responsibility between four emission sectors.

Emission Sector	S_1_	S_2_	S_3_	S_4_
Carbon Emission (*C_i_*) (million tonnes)	7118	7254	14,653	7728
Shared responsibility (*SR_i_*)	19%	20%	40%	21%

**Table 6:** The shared responsibility between sub-sectors.

Emission Sector	S_11_	S_12_	S_21_	S_22_	S_23_	S_24_	S_31_	S_32_	S_33_	S_34_	S_41_	S_42_	S_43_	S_44_	S_45_
Carbon Emission (*C_i_*) (million tonnes)	5654	1462	1621	1140	1314	3177	9054	2103	446	3078	5865	123	737	818	166
Shared responsibility (*SR_i_*)	79%	21%	22%	16%	18%	44%	62%	14%	3%	21%	76%	2%	9%	11%	2%

**Table 7:** Proportion of carbon emission in main countries and regions.

Country	2004	2005	2006	2007	2008	2009	2010	2011	2012	2013
China	18.6%	19.7%	21.1%	21.8%	22.4%	24.0%	26.2%	27.9%	28.3%	28.6%
United States	20.3%	19.7%	18.7%	18.6%	17.6%	16.6%	16.1%	15.2%	14.4%	14.5%
European Union	14.3%	13.7%	13.3%	12.8%	12.2%	11.4%	11.1%	10.2%	9.8%	9.5%
India	4.1%	4.2%	4.3%	4.5%	4.9%	5.5%	5.1%	5.3%	5.7%	5.7%
Russian Federation	5.7%	5.5%	5.5%	5.4%	5.4%	5.0%	5.0%	5.1%	5.2%	5.0%
Japan	4.5%	4.2%	4.0%	4.0%	3.8%	3.5%	3.5%	3.4%	3.5%	3.5%
Total	67.5%	67.0%	66.9%	67.1%	66.3%	66.0%	67.0%	67.1%	66.9%	66.8%
World	100.0%	100.0%	100.0%	100.0%	100.0%	100.0%	100.0%	100.0%	100.0%	100.0%

**Table 8:** Work plan for promoting low-carbon city.

No	Sample City	Country	Low-Carbon Work Plan	the Authority for Action
1	Beijing	China	Energy conservation and climate action plan [45]	Beijing Municipal Government
2	Shanghai	China	Thirteenth five-year plan of economic and social development [46]	Shanghai Municipal Government
3	Hong Kong	China	Hong Kong’s climate action plan 2030 [47]	Hong Kong Environment Bureau
4	Shenzhen	China	Mid-long term plans of low-carbon development [48]	Shenzhen Development and Reform Commission
5	Wuhan	China	Action plan of low-carbon city pilot [49]	Wuhan Municipal Government
6	New York	United States	Climate action plan interim report [50]	New York State Climate Action Council
7	San Francisco	United States	Climate action plan for San Francisco [51]	San Francisco Department of the Environment, San Francisco Public Utilities Commission
8	Los Angeles	United States	Unincorporated Los Angeles county community climate action plan 2020 [52]	County of Los Angeles, Department of Regional Planning
9	Chicago	United States	Chicago climate action plan [53]	City of Chicago
10	Philadelphia	United States	Local action plan for climate change [54]	City of Philadelphia, Sustainability Working Group
11	Austin	United States	Austin community climate plan [55]	City of Austin, Office of Sustainability
12	Seattle	United States	Seattle climate action plan [56]	Seattle Office of Sustainability & Environment
13	Portland	United States	Climate action plan [57]	City of Portland
14	London	England	A low-carbon London: now and beyond [58]	London sustainable development commission
15	Berlin	Germany	Climate-Neutral Berlin 2050 [59]	Senate Department for Urban Development and the Environment
16	Milan	Italy	Sustainable energy and climate action plan municipality of Milan [60]	Municipality of Milan Council of Environment
17	Amsterdam	Netherlands	Amsterdam: a different energy [61]	City of Amsterdam
18	Rotterdam	Netherlands	Rotterdam program on sustainability and climate change 2015–2018 [62]	City of Rotterdam
19	Copenhagen	Denmark	Copenhagen climate plan [63]	City of Copenhagen, Technical and Environmental Administration
20	Stockholm	Sweden	Stockholm action plan for climate and energy 2010–2020 [64]	Environment and Health Department
21	Madrid	Spain	City of Madrid energy and climate change action plan [65]	Energy Agency of Madrid
22	Delhi	India	Climate change agenda for Delhi 2009–2012 [66]	Chief Secretary Delhi
23	Tokyo	Japan	Tokyo climate change strategy: progress report and future vision [67]	Tokyo Metropolitan Government
24	Yokohama	Japan	Yokohama city action plan for global warming countermeasures [68]	Yokohama Climate Change Policy Headquarters

**Table 9:** Guidelines for promoting low-carbon city.

No	Guidelines for Low-Carbon City	Issuing Authority/Authors
1	Convenient Solutions to an Inconvenient Truth: Approaches to Climate Change [69]	World Bank
2	Low-Carbon City Development Program Guidebook: A Systems Approach to Low-Carbon Development in Cities [70]	World Bank
3	Low-Carbon City: A Guidebook for City Planners and Practitioners [71]	UNEP
4	Developing Local Climate Change Plans: a Guide for Cities in Developing Countries [72]	UN-Habitat
5	Roadmap 2050—A Practical Guide to A Prosperous, Low-carbon Europe [73]	European Climate Foundation
6	Low-Carbon City Policy Data book: 72 Policy Recommendations for Chinese Cities from the Benchmarking and Energy Savings Tool for Low Carbon Cities [74]	Price et al.
7	Integrated energy and carbon modeling with a decision support system: Policy scenarios for low-carbon city development in Bangkok [20]	Phdungsilp
8	Marginal abatement cost and carbon reduction potential outlook of key energy efficiency technologies in China’s building sector to 2030 [17]	He et al.
9	Mitigation from a cross-sectoral perspective [75]	Baker et al.

**Table 10:** Typical carbon reduction policies in different emission sectors.

	S_1_: Building	S_2_: Industry	S_3_: Energy transformation	S_4_: Transportation
MP	S_1_-MP_1_: Energy efficiency performance standards in new building S_1_-MP_2_: Energy efficiency performance standards of building appliance S_1_-MP_3_: Auditing reports of building energy-efficiency S_1_-MP_4_: Quota management of energy consumption S_1_-MP_5_: Retrofitting public building with energy-saving facility S_1_-MP_6_: Replacement of energy-saving lamp S_1_-MP_7_: Adoption of water cooling towers instead of air-conditioning systems S_1_-MP_8_: Replacement of obsolete water main S_1_-MP_9_: District heating network	S_2_-MP_1_: Energy efficiency standards of various industrial sectors S_2_-MP_2_: Application of advanced industrial equipment S_2_-MP_3_: Energy audits and assessments S_2_-MP_4_: Eliminating high-emission industries S_2_-MP_5_: Standards of emission in industrial processes S_2_-MP_6_: Adoption of advanced process technologies S_2_-MP_7_: Emission capture and destruction S_2_-MP_8_: Mandatory carbon reduction targets for industry	S_3_-MP_1_: Efficiency standards for power generators S_3_-MP_2_: Mandatory transformer upgrade program S_3_-MP_3_: District heating networking maintenance and upgrade program S_3_-MP_4_: Program of recuperating waste heat S_3_-MP_5_: Distributed electricity generation S_3_-MP_6_: Phasing down coal for electricity generation S_3_-MP_7_: Wind power generation program S_3_-MP_8_: Solar power generation program S_3_-MP_9_: Nuclear electric power generation S_3_-MP_10_: Tidal power Generation S_3_-MP_11_: Hot springs power generation and hot springs heat pump S_3_-MP_12_: Hydroelectric generation S_3_-MP_13_: Solar heating program S_3_-MP_14_: Hydrogen fuel cells S_3_-MP_15_: Bioenergy displace heating fuels	S_4_-MP_1_: Transit-oriented transportation planning S_4_-MP_2_: Mixed land uses to minimizes daily transfer distance S_4_-MP_3_: Enhancing the quality of public transport services S_4_-MP_4_: Bus rapid transit network S_4_-MP_5_: Improving complementarity of public transport S_4_-MP_6_: Rationalization of bus routes S_4_-MP_7_: Improving the operation efficiency of tramways S_4_-MP_8_: Developing intercity rail to foster more efficient freight movement S_4_-MP_9_: Extension of rail-lines S_4_-MP_10_: Standards of vehicle fuel using efficiency S_4_-MP_11_: Standards of vehicle carbon emission S_4_-MP_12_: Restriction on private car S_4_-MP_13_: Improving walk and bicycle path environment S_4_-MP_14_: Electronic toll collection system
EP	S_1_-EP_1_: Energy efficiency market for existing building S_1_-EP_2_: Green building labeling program and information disclosure S_1_-EP_3_: Financial support for energy service companies S_1_-EP_4_: Subsidies and tax credits for weatherization S_1_-EP_5_: Subsidies for purchasing energy-efficient equipment S_1_-EP_6_: Energy efficiency labelling for the major electrical appliances S_1_-EP_7_: Trade-in of energy-saving appliance S_1_-EP_8_: Appliance of smart consumption meters in residential buildings S_1_-EP_9_: Time-zone mechanism for electricity price	S_2_-EP_1_: Tax relief on carbon reduction projects S_2_-EP_2_: Provision of loans and funds for improving industrial energy efficiency and adopting innovative technologies S_2_-EP_3_: Carbon cap-and-trade program S_2_-EP_4_: Supporting energy management service companies S_2_-EP_5_: Carbon labelling scheme for industrial products S_2_-EP_6_: Subsidizing energy-efficient equipment	S_3_-EP_1_: Subsidies and tax incentives for renewable energy S_3_-EP_2_: Certification system for photovoltaic power generation equipment installers	S_4_-EP_1_: Financial incentives for the purchase of low-carbon vehicles. S_4_-EP_2_: Parking fees S_4_-EP_3_: Increase of fuel tax S_4_-EP_4_: Congestion charges
VP	S_1_-VP_1_: Energy conservation training for building maintenance staff S_1_-VP_2_: Public education on improving building energy efficiency S_1_-VP_3_: Expedited permitting for green buildings S_1_-VP_4_: Encouraging large building participate in climate initiative program S_1_-VP_5_: Demonstrative projects of ultra-low energy consumption building S_1_-VP_6_: Encourage solar installation S_1_-VP_7_: Encourage retrofit buildings with solar photovoltaics	S_2_-VP_1_: Encouraging larger companies to optimize manufacturing techniques S_2_-VP_2_: Encouraging companies to upgrade industrial equipment S_2_-VP_3_: Energy-saving technology services to industrial companies S_2_-VP_4_: Workforce training of energy saving in industrial sector S_2_-VP_5_: Demonstrative projects of low-carbon industry parks	S_3_-VP_1_: Encouraging larger companies to optimize operation management of power plant	S_4_-VP_1_: Publicity about saving energy on trip S_4_-VP_2_: Energy saving guidance for transportation companies S_4_-VP_3_: Publicity about clean-fuels vehicles S_4_-VP_4_: Promoting car-sharing programs S_4_-VP_5_: Encouragement of telecommuting work

MP: Mandatory Administration Policy; EP: Economic Incentive Policy; VP: Voluntary Scheme Policy.

**Table 11:** Number of cities in applying emission reduction policies.

Policy	*N_ij_*	Policy	*N_ij_*	Policy	*N_ij_*	Policy	*N_ij_*
S_1_-MP_1_	23	S_2_-MP_1_	2	S_3_-MP_1_	4	S_4_-MP_1_	13
S_1_-MP_2_	4	S_2_-MP_2_	1	S_3_-MP_2_	2	S_4_-MP_2_	6
S_1_-MP_3_	7	S_2_-MP_3_	2	S_3_-MP_3_	2	S_4_-MP_3_	11
S_1_-MP_4_	2	S_2_-MP_4_	5	S_3_-MP_4_	6	S_4_-MP_4_	6
S_1_-MP_5_	2	S_2_-MP_5_	1	S_3_-MP_5_	4	S_4_-MP_5_	9
S_1_-MP_6_	12	S_2_-MP_6_	2	S_3_-MP_6_	6	S_4_-MP_6_	2
S_1_-MP_7_	1	S_2_-MP_7_	2	S_3_-MP_7_	16	S_4_-MP_7_	2
S_1_-MP_8_	2	S_2_-MP_8_	1	S_3_-MP_8_	19	S_4_-MP_8_	4
S_1_-MP_9_	4	S_2_-EP_1_	1	S_3_-MP_9_	2	S_4_-MP_9_	6
S_1_-EP_1_	1	S_2_-EP_2_	2	S_3_-MP_10_	4	S_4_-MP_10_	15
S_1_-EP_2_	2	S_2_-EP_3_	4	S_3_-MP_11_	2	S_4_-MP_11_	4
S_1_-EP_3_	2	S_2_-EP_4_	4	S_3_-MP_12_	2	S_4_-MP_12_	7
S_1_-EP_4_	11	S_2_-EP_5_	2	S_3_-MP_13_	5	S_4_-MP_13_	18
S_1_-EP_5_	5	S_2_-EP_6_	6	S_3_-MP_14_	2	S_4_-MP_14_	7
S_1_-EP_6_	6	S_2_-VP_1_	5	S_3_-MP_15_	11	S_4_-EP_1_	9
S_1_-EP_7_	4	S_2_-VP_2_	4	S_3_-EP_1_	4	S_4_-EP_2_	6
S_1_-EP_8_	4	S_2_-VP_3_	3	S_3_-EP_2_	2	S_4_-EP_3_	2
S_1_-EP_9_	4	S_2_-VP_4_	4	S_3_-VP_1_	2	S_4_-EP_4_	6
S_1_-VP_1_	2	S_2_-VP_5_	2			S_4_-VP_1_	6
S_1_-VP_2_	18					S_4_-VP_2_	4
S_1_-VP_3_	1					S_4_-VP_3_	15
S_1_-VP_4_	2					S_4_-VP_4_	13
S_1_-VP_5_	1					S_4_-VP_5_	6
S_1_-VP_6_	15						
S_1_-VP_7_	7						

**Table 12:** Contributed efforts in four emission sectors.

Sector	S_1_: Building	S_2_: Industry	S_3_: Energy Transformation	S_4_: Transportation
*CE_i_’*	295	104	275	420
*CE_i_*	27%	10%	25%	38%

**Table 13:** The number of three kinds of policies adopted in four emission sectors.

Sector	MP	EP	VP	Total
S_1_: Building	57	39	46	142
S_2_: Industry	16	19	18	53
S_3_: Energy Transformation	87	6	2	95

## Appendix A

**Table A1:** Energy consumption by various sectors from WEB.

Sector	Sub-Sector	Coal	Crude Oil	Oil Products	Natural Gas	Biofuels	Heat	Electricity
Transformation Process and Energy industry	E1. Transfers	0.67	292.66	−330.35	0.00	0.00	0.00	0.00
	E2. Statistical differences	31.30	−0.17	−6.44	−20.97	−0.23	0.64	0.61
	E3. Electricity plants	3018.60	58.03	288.42	1101.55	135.76	1.03	−2174.81
	E4. CHP plants	235.16	0.01	24.39	439.34	82.04	−211.88	−256.73
	E5. Heat plants	186.18	0.97	18.84	112.60	16.36	−256.68	0.54
	E6. Blast furnaces	299.78	0.00	0.54	0.23	0.07	0.00	0.00
	E7. Gas works	15.60	0.00	3.90	−7.26	0.13	0.00	0.00
	E8. Coke/pat.fuel/BKB/PB plants	108.93	0.00	4.00	0.01	0.17	0.00	0.00
	E9. Oil refinries	0.00	5890.16	−5785.26	0.00	0.00	0.00	0.00
	E10. Petrochemical plants	0.00	−47.14	46.60	0.00	0.00	0.00	0.00
	E11. Liquefaction plants	13.81	−20.04	0.00	24.89	0.00	0.00	0.00
	E12. Other transformation	0.61	−14.39	0.74	16.97	118.43	1.04	0.00
	E13. Energy industry own use	145.37	16.31	293.28	416.71	19.91	49.73	203.14
	E14. Losses	5.56	12.71	0.93	31.10	0.27	27.97	241.85
Transport	E15. World aviation bunkers	0.00	0.00	240.69	0.00	0.00	0.00	0.00
	E16. Domestic aviation	0.00	0.00	153.60	0.00	0.00	0.00	0.00
	E17. Road	0.00	0.00	2663.84	54.43	104.46	0.00	0.31
	E18. Rail	4.01	0.00	42.37	0.00	0.36	0.00	23.22
	E19. Pipeline transport	0.00	0.00	0.50	84.29	0.00	0.00	3.89
	E20. World marine bunkers	0.00	0.00	278.06	0.00	0.11	0.00	0.00
	E21. Domestic navigation	0.00	0.00	76.22	0.16	0.61	0.00	0.00
	E22. Non-specified	0.07	0.00	10.99	1.00	0.01	0.00	4.41
Industry	E23. Iron and steel	470.90	0.00	1.01	79.06	5.00	22.10	118.02
	E24. Chemical and petrochemical	142.00	0.09	78.57	172.95	2.33	71.77	117.34
	E25. Non-ferrous metals	34.69	0.00	7.10	24.00	0.09	4.79	113.76
	E26. Non-metallic minerals	346.61	0.01	59.29	78.22	12.96	4.46	60.27
	E27. Transport equipment	5.19	0.00	2.94	17.04	0.07	5.77	33.70
	E28. Machinery	20.56	0.00	10.30	36.73	0.23	7.64	112.25
	E29. Mining and quarrying	14.69	0.00	32.87	10.29	0.24	3.30	42.17
	E30. Food and tobacco	46.00	0.01	15.60	64.60	44.03	15.73	57.87
	E31. Paper pulp and printing	27.19	0.00	6.39	33.21	87.40	17.00	48.46
	E32. Wood and wood products	5.19	0.00	2.96	4.14	10.84	2.89	14.57
	E33. Construction	6.94	0.00	41.16	9.70	0.47	1.91	21.46
	E34. Textile and leather	19.93	0.01	5.74	8.91	0.39	9.94	41.02
	E35. Non-specified	86.57	9.59	147.02	101.90	112.42	8.44	188.19
Other	E36.Residential	107.22	0.00	295.83	599.53	1210.75	150.45	535.91
	E37.Commercial and public services	49.96	0.00	122.15	259.61	34.99	50.36	437.94
